# Retraction Note: The association between early career informal mentorship in academic collaborations and junior author performance

**DOI:** 10.1038/s41467-020-20617-y

**Published:** 2020-12-21

**Authors:** Bedoor AlShebli, Kinga Makovi, Talal Rahwan

**Affiliations:** 1 Department of Computer Science, Science Division, New York University Abu Dhabi, Abu Dhabi, UAE; 2 Computational Social Science Lab, Social Science Division, New York University Abu Dhabi, Abu Dhabi, UAE; 3 Social Research and Public Policy, Social Science Division, New York University Abu Dhabi, Abu Dhabi, UAE

**Keywords:** Scientific community, Careers

Retraction of: *Nature Communications* 10.1038/s41467-020-19723-8, published online 17 November 2020.

The Authors are retracting this Article in response to criticisms about the assumptions underpinning the Article in terms of the identification of mentorship relationships and the measure of mentorship quality, challenging the interpretation of the conclusions. These criticisms were raised by readers and confirmed by three experts post-publication as part of a journal-led investigation.

In this Article, we analysed publication records to identify pairs of junior and senior researchers working in the same discipline, at the same institution, who are co-authors on papers with no more than 20 authors. We use co-authorship, as defined above, as a proxy of mentorship by senior researchers, with the support of a survey that was targeted at a random sample of a recent cohort of researchers. We measure the quality of mentorship using the number of citations and the connectedness of the senior investigators.

The three independent experts commented on the validity of the approaches and the soundness of the interpretation in the Article. They supported previous criticisms in relation to the use of co-authorship as a measure of mentorship. Thus, any conclusions that might be drawn on biases in citations in the context of co-authorship cannot be extended to informal academic mentorship. The experts also noted that the operationalisation of mentorship quality was not validated in the paper.

Although we believe that all the key findings of the paper with regards to co-authorship between junior and senior researchers are still valid, given the issues identified by reviewers about the validation of key measures, we have concluded that the most appropriate course of action is to retract the Article.

We are an interdisciplinary team of scientists with an unwavering commitment to gender equity, and a dedication to scientific integrity. Our work was designed to understand factors that influence the scientific impact of those who advance in research careers. We feel deep regret that the publication of our research has both caused pain on an individual level and triggered such a profound response among many in the scientific community. Many women have personally been extremely influential in our own careers, and we express our steadfast solidarity with and support of the countless women who have been a driving force in scientific advancement. We hope the academic debate continues on how to achieve true equity in science–a debate that thrives on robust and vivid scientific exchange.

All Authors agree with the retraction.

